# Toward Accurate yet Effective Computations of Rotational Spectroscopy Parameters for Biomolecule Building Blocks

**DOI:** 10.3390/molecules28020913

**Published:** 2023-01-16

**Authors:** Vincenzo Barone, Silvia Di Grande, Cristina Puzzarini

**Affiliations:** 1 Scuola Normale Superiore, Piazza dei Cavalieri 7, I-50126 Pisa, Italy; 2 Scuola Superiore Meridionale, Largo San Marcellino 10, I-80138 Napoli, Italy; 3 Rotational and Computational Spectroscopy Lab, Department of Chemistry “Giacomo Ciamician”, University of Bologna, Via F. Selmi 2, I-40126 Bologna, Italy

**Keywords:** biomolecule building blocks, quantum-chemical composite schemes, double-hybrid density functional, rotational spectroscopy

## Abstract

The interplay of high-resolution rotational spectroscopy and quantum-chemical computations plays an invaluable role in the investigation of biomolecule building blocks in the gas phase. However, quantum-chemical methods suffer from unfavorable scaling with the dimension of the system under consideration. While a complete characterization of flexible systems requires an elaborate multi-step strategy, in this work, we demonstrate that the accuracy obtained by quantum-chemical composite approaches in the prediction of rotational spectroscopy parameters can be approached by a model based on density functional theory. Glycine and serine are employed to demonstrate that, despite its limited cost, such a model is able to predict rotational constants with an accuracy of 0.3% or better, thus paving the way toward the accurate characterization of larger flexible building blocks of biomolecules.

## 1. Introduction

The experimental study of biomolecule building blocks in the gas phase has recently attracted increasing attention owing to the development of spectrometers coupling supersonic-jet expansion [1] with laser ablation [2]. This has allowed the application of high-resolution rotational spectroscopy in the microwave region to thermolabile molecules with high melting points. Among these, we find the main families of life bricks such as amino acids, peptides, DNA and RNA bases. The focus of the present contribution is the computational support to rotational spectroscopy investigations of building blocks of biomolecules.

For flexible molecules such as those of interest for this study, a great challenge is related to the large number of conformers and the fast relaxation of some of them to more stable counterparts due to the presence of low interconversion energy barriers. An inaccurate account of the relaxation processes can bias any direct thermochemical interpretation of the results provided by rotational spectroscopy experiments [3,4]. Quantum-chemical (QC) computations can help to tackle this challenge, especially because gas phase is their most natural playground [5,6]. Unfortunately, already for medium-sized systems, the usual dichotomy between accuracy and feasibility, which is the quest for accurate yet feasible predictions, comes into place [7]. In fact, state-of-the-art QC approaches are able to rival the experimental counterparts for small semi-rigid systems in the gas phase [7,8,9], but they are characterized by a very unfavorable scaling with the dimension of the system to be investigated. This prevents their brute-force application already to biomolecule building blocks containing more than a dozen atoms and characterized by several low-energy minima. Furthermore, the powerful local optimization techniques developed for semi-rigid systems are not effective for flexible systems, which requires the exploration of rugged potential energy surfaces (PESs) [10,11].

For the reasons mentioned above, the accurate characterization needed by rotational spectroscopy requires an integrated computational approach that employs QC models of increasing accuracy in the different steps of an exploration/exploitation strategy guided by machine learning (ML) tools. The main steps of this strategy [10,12,13,14] can be summarized as follows:

1. Unsupervised perception of the molecular system to identify hard and soft degrees of freedom [15];
2. Knowledge-based selection and constrained geometry optimizations of a limited number of conformers employing a fast semi-empirical method [11,16];
3. Exploration of the PES governed by soft degrees of freedom using the same semi-empirical method of the previous step, guided by a purposely tailored evolutionary algorithm with the aim of finding other low-lying minima [10];
4. Refinement of the most stable structures by hybrid and then double-hybrid density functionals [14];
5. Analysis of relaxation paths between pairs of adjacent energy minima [13];
6. Evaluation of accurate electronic energies for the final panel of low-energy minima [17,18,19];
7. Computation of zero point energies (ZPE) and thermal contributions to enthalpies and entropies [20,21,22,23,24,25];
8. Computation of spectroscopic parameters for the energy minima with non negligible populations [13,26].

The focus of the present contribution is on points 6 to 8. First, we will validate the accuracy of approximate approaches based on double-hybrid functionals and effective composite methods by exploiting results available for semi-rigid molecules and non-covalent complexes. Next, amino acids will be employed to illustrate the potentialities of the validated approaches. These compounds represent a particularly appealing playground because their rich conformational landscape is tuned by the competition among different types of intra-molecular non-covalent interactions involving, together with the amino and carboxylic acid moieties of the backbone, also side-chain groups. At the same time, results from MW experiments are available for several conformers of most natural α-amino acids [27,28,29,30,31,32,33,34,35,36,37,38], and provide accurate data for benchmarking theory. Among the α-amino acids well characterized experimentally, we have selected glycine and serine.

Since the current standard for the computation of rotational spectroscopy parameters of biomolecule building blocks (see, for example, [4,27,39,40,41]) is based on QC methodologies of limited accuracy and does not account for vibrational effects, together with the intrinsic interest of the studied molecules, the results of the present study will provide a reference for a more accurate and reliable approach to be used in the prediction and analysis of MW experiments.

## 2. Results and Discussion

### 2.1. The Methodologic Approach

Several studies have shown that the double-hybrid rev-DSD-PBEP86 functional [42] in conjunction with the jun-cc-pVTZ [43] basis set (this functional-basis combination is shortly denoted as rDSD in the following) represents a very robust and convenient choice for obtaining equilibrium geometries suitable for subsequent electronic energy evaluations [19,44,45,46]. For the latter, composite schemes rooted in the coupled-cluster (CC) theory [47] that account for the extrapolation to the complete basis set (CBS) limit and core–valence (CV) correlation are employed whenever aiming at high accuracy [13,25,46,48,49,50]. However, composited schemes entirely based on CC theory are computationally expensive. The key idea of the so-called ‘cheap’-scheme (ChS) family of composite approaches [17,18,19,51] is that both CBS and CV contributions can be estimated accurately by low-order (hence low-cost) many-body perturbative methods, with Møller–Plesset theory to second order (MP2) [52] offering the best compromise between accuracy and computational cost. The general expression of the electronic energy evaluated using the ChS model is:

(1) $$E_{\mathrm{ChS}}=E\left(\mathrm{CCSD}\left(T\right)/\mathrm{TZ}\right)+\Delta E\left(\mathrm{MP}2/\mathrm{CBS}\right)+\Delta E\left(\mathrm{MP}2/\mathrm{CV}\right)\;,$$

where the first term on the right-hand side is the energy at the fc-CCSD(T) [53] level in conjunction with a triple-zeta (TZ) basis set, where CCSD(T) denotes the CC method incorporating single, double, and a perturbative estimate of triple excitations, and fc stands for the frozen-core approximation. The second term is the contribution due to the extrapolation to the CBS limit evaluated at the MP2 level within the fc approximation and using the $n^{-3}$ formula [54]:

(2) $$\Delta E\left(\mathrm{MP}2/\mathrm{CBS}\right)=\frac{n^{3}E_{\mathrm{MP}2/nZ}-{\left(n-1\right)}^{3}E_{\mathrm{MP}2/(n-1)Z}}{n^{3}-{\left(n-1\right)}^{3}}-E_{\mathrm{MP}2/(n-1)Z}\;,$$

where *n* = 4 (quadruple-zeta basis set, QZ) and *n*-1 stands for TZ. The last term, ΔE(MP2/CV), is the CV contribution computed as energy difference between MP2 calculations correlating all electrons and within the fc approximation, both in the cc-p(w)CVTZ basis set [55,56].

The first implementation of the ChS model employed the cc-pVnZ basis sets [57]. However, several benchmarks [18,19] have led to the conclusion that the best cost/performance ratio is obtained with the jun-cc-pVnZ basis sets [43], with jun-cc-pV(n+*d*)Z being used for atoms of the third period [58]. This model, hereafter referred to as junChS, has become our standard ChS methodology. A more recent alternative [19] replaces the conventional CCSD(T) and MP2 methods with their explicitly-correlated counterparts, namely CCSD(T)-F12 and MP2-F12 [59,60], still in conjunction with the jun-cc-pVnZ and cc-p(w)CVTZ basis sets. Hereafter, this model is referred to as junChSF12. In all CCSD(T)-F12 computations, the F12b approximation [61] is employed, while the default approximation (3C/FiX) is used for MP2-F12 [60].

In addition to energy evaluations, different ChS variants have been employed and tested for geometry optimizations [19,51,62,63]. For conventional QC methods, ChS and junChS are defined analogous to Equations (1) and (2), whereas the extrapolation to the CBS limit (but not the CV correction) can be avoided (and sometimes introduces non-negligible oscillations) for approaches exploiting explicitly-correlated methods. The models derived and employed in the present work are denoted as DZCCF12+CV, TZCCF12+CV, junCCF12+CV and augCCF12+CV, which correspond to CCSD(T)-F12 calculations in conjunction with cc-pVDZ-F12, cc-pVTZ-F12, jun-cc-pVTZ and aug-cc-pVTZ, respectively. The “+CV” denotes the incorporation of the CV contribution, which is evaluated at the MP2-F12/cc-p(w)CVTZ level.

When dealing with flexible molecules, a key point is the determination of the relative stability of the low-energy minima to determine those that are sufficiently populated for a spectroscopic characterization. To accomplish this task, one has to move from electronic energy differences to the corresponding relative enthalpies or free energies, evaluated at a temperature that depends on the experimental conditions. To this end, ZPEs and vibrational partition functions are needed. Within the harmonic approximation, this task requires the evaluation of harmonic vibrational frequencies, which can be obtained with a suitable accuracy at the rDSD level [64] exploiting analytical second derivatives [65]. The models of the ChS family can also be applied to the computation of harmonic frequencies. However, due to effective error compensation, improved results are obtained by neglecting the CV correction for explicitly-correlated approaches [66]. Focusing on models that exploit the F12 methodology, the DZCCF12, TZCCF12, junCCF12 and augCCF12 approaches offer a good compromise between accuracy and computational cost. The ZPE term can then be improved by incorporating anharmonic corrections, which are usually obtained at the B3LYP/jun-cc-pVDZ level (hereafter B3) within second-order vibrational perturbation theory (VPT2, [20,67]). Whenever required, the contribution of low-frequency motions to entropies can be computed using the quasi-harmonic (QH) approximation [24,68]. Within the QH approximation, below a given cut-off value, entropic terms are obtained from the free-rotor approximation, and a damping function is used to interpolate between free-rotor and harmonic oscillator expressions close to the cut-off frequency.

Moving to rotational spectroscopy, the leading terms are the rotational constants, which incorporate two contributions. The first, and by far the largest, one is the equilibrium rotational constant, B${}_{e}$, which is straightforwardly derived from the equilibrium geometry of the studied molecule. The second contribution is related to vibrational effects which are present because of the coupling between vibrations and rotation. This contribution is usually approximated by means of VPT2, which, for the vibrational gound state, leads to:

(3) $$B_{0}^{i}=B_{e}^{i}-\frac{1}{2}\sum_{r}\alpha_{r}^{i}=B_{e}^{i}+\Delta B_{vib}^{i}\;.$$

where $\alpha_{r}^{i}$ denotes the vibrational-rotation interaction constants, with *i* being the inertial axis (a, b, or c) and the sum running over all the *r* vibrational modes. The computation of this contribution requires both second- and semi-diagonal third-energy derivatives of the energy with respect to normal modes. Therefore, computations of vibrational corrections to rotational constants are quite expensive. As a consequence, its inclusion is warranted only if the error on the computed equilibrium rotational constants is smaller than the expected vibrational contribution. A recent careful analysis of these aspects provides some general hints [69]. The magnitude of the vibrational contribution ΔB${}_{vib}$ is typically 0.1% to 0.7% that of the corresponding equilibrium rotational constant, with 0.5% being a very robust guess for semi-rigid molecules. As a consequence, an uncertainty of 10% on ΔB${}_{vib}$ would lead to an error of 0.05% on the rotational constants, which is thus more than acceptable. The overall conclusion is the suitability of global hybrid density functionals in conjunction with double-zeta basis sets (e.g., the B3 model previously defined) for the computation of the required anharmonic force fields, thus largely reducing the computational cost.

Focusing on the accuracy needed by rotational spectroscopy, errors of 1% on rotational constants (100 MHz for a constant with magnitude 10 GHz) are not at all helpful for the prediction and/or analysis of rotational spectra of flexible molecules, which are always characterized by several low-energy minima with non negligible populations. On the other hand, an accuracy of 0.01%, which would allow direct comparison with experiment, is presently reachable only for molecules containing two or three atoms. Based on these considerations, the optimal level of accuracy associated with predicted rotational constants should be close to 0.1% (10 MHz for a constant of 10 GHz). In terms of structural parameters, such an accuracy corresponds to errors smaller than 0.001 Å for typical bond lengths and 0.001 radians (0.05 degrees) for typical valence angles [69]. This target accuracy can be surely obtained by expensive composite schemes incorporating high excitation orders in the correlation treatment [70]. Nevertheless, the models of the ChS family are able to draw closer to such a high accuracy limit [51,62,69]. However, for quite large flexible molecules, even this computational level becomes too expensive and one has to rely on density functional theory (DFT).

To improve the optimized geometries obtained from double-hybrid functionals, one can resort to the the so-called linear regression approach (LRA) [71,72,73,74]. This corrects the computed bond lengths ($r_{comp}$) for systematic errors by means of scaling factors (a) and offset values (b) that have been derived from the comparison of DFT and accurate semi-experimental (SE) equilibrium geometries for a large database:

(4) $$r=\left(1+a\right)\times r_{comp}+b\;,$$

with the a and b parameters depending not only on the functional considered, but also on the nature of the atoms involved. These are available in [73].

As mentioned above, the equilibrium rotational constants contribute more than 99% to the parameters derived from experiments and only depend on the molecular structure and isotopic composition [75]. Indeed, rotational constants are inversely proportional to the inertia tensor, which contains information on the mass distribution in the molecule. Therefore, a comparison between computed and experimental rotational constants can provide hints on the quality of the computed structure. In this respect, additional parameters of particular relevance are the nuclear quadrupole coupling constants ($\chi_{ii}$, *i* referring to the inertia axis a, b or c), which strongly depend on the intra-molecular interactions [76]. Nuclear quadrupole coupling is the interaction between the quadrupole moment of a quadrupolar nucleus and the electric gradient at the nucleus itself, with quadrupolar nuclei being those having a nuclear spin I≥1 [75]. Since ${}^{14}$N is a quadrupolar nucleus and is present in almost all biomolecule building blocks, nuclear quadrupole coupling constants can be used, together with rotational constants, to assess the quality of computed structures. In turn, their calculation is important for accurate predictions of rotational spectra because nuclear quadrupole coupling determines a splitting of the rotational energy levels, thus causing a splitting of the rotational transitions, which is the so-called hypefine structure. It is also noted that vibrational effects on nuclear quadrupole coupling constants are usually smaller than the uncertainty affecting the computed equilibrium values, and thus have not been considered in this work.

As far as technical details are concerned, all DFT, MP2 and single-point energy CCSD(T) computations have been performed using the Gaussian program package [77], while the CFOUR program [78] has been employed for geometry optimizations at the CCSD(T) level. For explicitly-correlated calculations, the MOLPRO program [79] has been used.

### 2.2. The Validation Step

Non-covalent complexes represent a suitable benchmark for the validation of computational methodologies aiming at an accurate description of intra-molecular non-covalent interactions. In a series of recent papers [18,19,80], different ChS variants have been applied to the computation of the interaction energies of prototypical systems. These studies (partially summarized in Table 1) show that the junChS and, especially, junChSF12 approaches allow the determination of interaction energies with an average error smaller than 10 cm${}^{-1}$ (0.12 kJ mol${}^{-1}$) without the need for incorporating any empirical parameter. In Table 1, the non-covalent complexes of the A14 database [19], which is a based on a selection of systems from the A24 dataset [81], have been considered. As reference values, the highly accurate data (which also incorporate up to quadruple excitation in the CC expansion and relativistic effects) from [82] have been selected.

From an inspection of Table 1, we note that going from junCCF12+CV to junChSF12, which only differ for the extrapolation to the CBS limit, leads to a significant lowering of both the maximum and mean unsigned error (MAX and MUE, respectively), with this confirming the importance of the extrapolation to the CBS limit also for explicitly-correlated methods and that this contribution can be accurately incorporated by means of low-order perturbative methods (here MP2-F12). Indeed, MP2-F12 represents a reliable route for obtaining accurate results without any significant computational increase with respect to the underlying CCSD(T)-F12 step.

Despite the extrapolation to the CBS limit, all ChS models (both conventional and F12 variants) are affected by small, but not entirely negligible basis set superposition errors (BSSE), which can be taken into account by means of counterpoise (CP) corrections (for details, the reader is referred to [19]). However, these are slightly more pronounced when considering the model based on conventional methods. This is particularly significant for intra-molecular interactions where the BSSE becomes ill defined and CP corrections difficult to implement. An important outcome of [18,19] is that, when using the rDSD level for the evaluation of the reference structures, non-CP corrected geometries can be safely employed. This is particularly important in view of applying junChS and junChSF12 to the evaluation of the energetics for flexible molecules. Concerning interaction energy, the errors at the rDSD level without any CP correction nearly double with respect to junChS(F12) [19], but they remain significantly smaller than those issued by other density functionals. More importantly, the rDSD level is suitable for defining a correct stability order for different kinds of non-covalent interactions.

As mentioned above, the largest contribution to rotational constants comes from their equilibrium value, which is related to the corresponding equilibrium geometry. In this connection, ChS, junChS and junChSF12 have proven to provide very accurate results [19,63], with the former two models being rather well tested [83]. When employing explicitly-correlated methods, the computational cost of the ChS methodology can be further reduced by omitting the CBS extrapolation step. To inspect this, a validation of the junChS and CCF12+CV models (for CCF12, we considered the DZCCF12, TZCCF12 and junCCF12 variants) has been performed by comparing their results with accurate SE equilibrium geometries from the SE100 database [73]. The subset considered includes 24 molecules containing hydrogen, second- and third-row atoms: CH${}_{4}$, CO${}_{2}$, HCN, HNC, H${}_{2}$O, NH${}_{3}$, C${}_{2}$H${}_{2}$, C${}_{2}$H${}_{4}$, H${}_{2}$CO, t-HCOOH, CH${}_{2}$NH, BH${}_{3}$NH${}_{3}$, BH${}_{2}$OH, C${}_{2}$H${}_{4}$O (oxirane), C${}_{2}$H${}_{4}$NH (aziridine), C${}_{3}$H${}_{6}$ (cyclopropane), H${}_{2}$O${}_{2}$, SO${}_{2}$, H${}_{2}$S, PH${}_{3}$, H${}_{2}$CS, CH${}_{2}$PH, C${}_{2}$H${}_{4}$S (tiirane) and H${}_{2}$S${}_{2}$. The results, collected in Table 2 in terms of maximum and mean unsigned errors (MAX, MUE), show that the CV contribution cannot be neglected and that the jun-cc-pVTZ basis set is competitive with its counterparts (cc-pVDZF12 and cc-pVTZF12) developed specifically for explicitly correlated models.

Even if not reported in Table 2, from [73], it is observed that—for the entire SE100 set—the rDSD model delivers remarkably accurate geometrical parameters with typical absolute errors of about 0.003 Å for bond lengths and 0.1–0.2 degrees for valence angles. Furthermore, the errors appear to be very systematic. Therefore, the rDSD level is particularly suitable for the application of the LRA mentioned above, which greatly reduces the expected errors [73]. In particular, in view of the focus of this work, the accuracy for the corresponding rotational constants becomes close to the 0.1% uncertainty-target.

The last issue touched in this validation step concerns the computation of reliable ZPEs and thermodynamic functions. As already mentioned, this requires accurate vibrational frequencies, whose leading terms are the corresponding harmonic values. For the purpose of finding the best compromise between accuracy and computational cost, we have computed the harmonic frequencies of eight molecules (hereafter referred to as the H8 set) for which either accurate experimental values are available (H${}_{2}$O [84], HCN [85], CO${}_{2}$ [86], C${}_{2}$H${}_{2}$ [87]) or whose computed force fields were adjusted in variational calculations for best agreement with experiment (H${}_{2}$CO [88], C${}_{2}$H${}_{2}$O [89], NH${}_{3}$ [90] and PH${}_{3}$ [91]). As already pointed out, CV contributions have not been considered.

As before, results are reported in terms of MAX, MUE and RMSD. From Table 3, it is quite apparent that the different basis sets tested in conjunction with the CCSD(T)-F12 ansatz provide very similar results. Therefore, the jun-cc-pVTZ basis set, which has been found to be the optimal choice for energy and geometry evaluations, can be confidently employed also for harmonic frequencies. Furthermore, the much cheaper rDSD level performs remarkably well and can be profitably used for the computation of harmonic frequencies of larger molecules and thus for the accurate evaluation of their ZPEs.

### 2.3. Amino Acids

**Figure 1:**
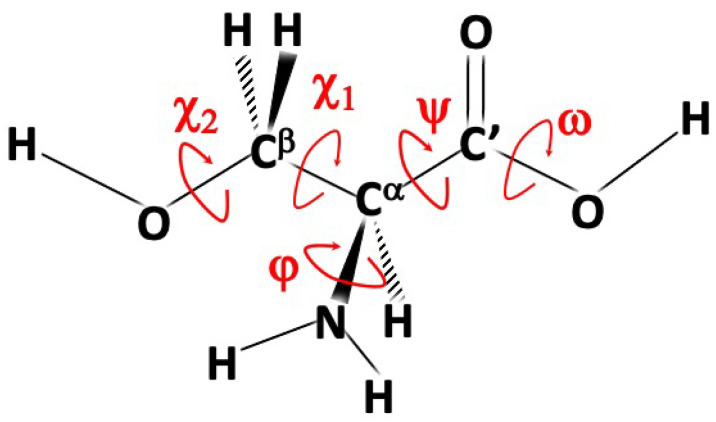
Structure and main dihedral angles of serine. See the main text for further details.

The structure of isolated amino acids is ruled by both backbone (ϕ = HNC${}^{\alpha }$C’, ψ = NC${}^{\alpha }$C’O(H) and ω = C${}^{\alpha }$C’OH) and side-chain (χ) torsional angles (see Figure 1 for the specific case of serine). The non-planarity of the NH${}_{2}$ moiety suggests that, instead of the customary ϕ dihedral angle, $\phi^{\prime }$ = LP-N-C${}^{\alpha }$-C’ = ϕ + 120${}^{\circ }$ can be better employed. In the definition above, LP is the nitrogen lone-pair perpendicular to the plane defined by the two aminic hydrogens and the C${}^{\alpha }$ atom.

**Figure 2:**
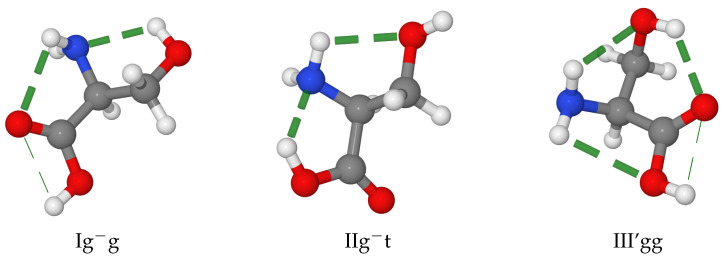
Representations of low-lying serine conformers of type I, II and III’. H-bonds are highlighted by dashed lines.

The most stable backbone structures result from the formation of hydrogen bonds, which can be classified as follows, also according to the values of the dihedral angles defined above. Type I is a bifurcated NH${}_{2}\cdots$O=C hydrogen bond ($\phi^{\prime }\approx 180^{\circ }$, $\psi \approx 180^{\circ }$, $\omega \approx 180^{\circ }$), type II is a N⋯HO hydrogen bond ($\phi^{\prime }\approx 0^{\circ }$, $\psi \approx 0^{\circ }$, $\omega \approx 0^{\circ }$), and type III is a bifurcated NH${}_{2}\cdots$OH hydrogen bond ($\phi^{\prime }\approx 180^{\circ }$, $\psi \approx 0^{\circ }$, $\omega \approx 180^{\circ }$). The type of hydrogen bond in the backbone also leads to the principal denomination of the structure, as shown in Figure 2. It has to be noted that type III structures have never been observed in MW experiments of natural α-aminoacids without polar side-chains due to their easy relaxation to the corresponding type I counterparts. Additional hydrogen bonds can be established with polar side chains, and they further stabilize the structure. This is the case, for example, of the type III’ conformers (single NH⋯OH hydrogen bond, $\phi^{\prime }\approx 180^{\circ }$, $\psi \approx 90^{\circ }$, $\omega \approx 180^{\circ }$). Typical conformers of types I, II and III’ are shown in Figure 2 for the case of serine (which will be discussed in detail in the following), where the g${}^{-}$, g and t labels refer to the gauche and trans conformations of each χ dihedral angle (as evident from Figure 1, for serine, there are two χ angles).

While the strategy described in the Introduction is able to identify all possible conformers of a given amino acid, their experimental characterization is limited by the sensitivity of the technique employed. For the specific case of rotational spectroscopy, a conservative limit for the relative stability of detectable structures is around 900 cm${}^{-1}$, which corresponds to a relative population of about 1% at room temperature (where kT/hc = 207 cm${}^{-1}$). In the same vein, an upper limit of 400 cm${}^{-1}$ for interconversion barriers is usually employed for discriminating fast relaxation processes in the case of flexible compounds showing several stable minima [4,39].

For the simplest amino acid, i.e., glycine, eight conformers have been characterized computationally, with only two of them (I and II) being sufficiently stable (and non-involved in fast relaxation processes) to be detected in MW experiments [92]. The limited size of glycine has allowed the evaluation of relative stabilities by means of state-of-the-art composite schemes including, together with CBS and CV contributions evaluated at the CCSD(T) level, full account of triple excitations, perturbative estimate of quadruple excitations, diagonal corrections to the Born–Oppenheimer approximation and relativistic effects [93]. While the reader is referred to [49,92,93,94] for details, here we point out the remarkable performance of the ChS and junChSF12 approaches in the evaluation of relative energies (Table 4), with MUEs smaller than 10 cm${}^{-1}$ with respect to the most accurate results [93]. Noticeably, the rDSD model performs well with a MUE of 15 cm${}^{-1}$. It is worth noting that such an accuracy is by far better than that obtainable by the methods (B3LYP and MP2) usually employed in the interpretation of MW spectra of biomolecule building blocks (see Table 4). Similar remarks apply to spectroscopic parameters. In fact, the MUEs with respect to experimental rotational constants are 23.6 MHz for rDSD, 13.0 MHz for rDSD-LRA, and 17.1 MHz for ChS. These average deviations from experiment point out the great accuracy that can be reached by applying LRA corrections to rDSD structures. For glycine, the availability of an accurate SE equilibrium structure for conformer I permits to evaluate directly the accuracy of geometrical parameters for the rDSD and rDSD-LRA levels. For rDSD, the MUEs are 0.0019 Å for bond lengths and 0.0026 radians (0.15 degrees) for valence angles, with the former value decreasing to 0.0004 Å at the rDSD-LRA level. Moving to nuclear quadrupole coupling constants, we note the good agreement (nearly quantitative) with experiment obtained by the ChS and rDSD levels, with the latter requiring a greatly reduced computational effort with respect to the former. This outcome further confirms the good performance of the rDSD model.

Systematic investigations have revealed that, analogous with glycine, the natural amino acids containing simple non-polar side chains (alanine [29], valine [30], isoleucine [31] and leucine [32]) have two dominant conformers of type I and II. On the contrary, the conformational landscape of natural amino acids with polar side chains is much richer due to the cooperativity or competition between intra-backbone and backbone with side-chain hydrogen bonds. The simplest amino acid containing a polar side-chain is serine, which has two degrees of freedom in its CH${}_{2}$OH side-chain: $\chi_{1}$ = N-C${}^{\alpha }$-C${}^{\beta }$-O and $\chi_{2}$ = C${}^{\alpha }-$C${}^{\beta }$-O-H (see Figure 1). The exploration of the conformational PES and refinement of low-lying energy minima end up with 12 structures within 900 cm${}^{-1}$ above the absolute energy minimum. However, five of them relax to more stable counterparts through low energy barriers. Thus, seven conformers are left as possibly detectable in MW experiments: three of type II, two of type III’, and one each for types I and I’. Contrary to the case of glycine, the most stable conformer of serine in terms of electronic energies is of type II. However, incorporation of ZPE and thermal effects reverses the situation. As a result, the most stable conformer is Ig${}^{-}$g in terms of standard free energies.

The rotational constants of the two most stable conformers have been recently computed by geometry optimizations at the ChS level, with relative MAX and MUE of 0.6% and 0.3% with respect to the experiment, respectively [95]. It is noteworthy that very similar (but slightly improved) relative errors (0.4% and 0.3%, respectively) are obtained at the rDSD-LRA level (see Table 4). The strongly reduced computational requirements of this level have allowed us to obtain accurate results also for the other five low-energy conformers experimentally characterized, the results being provided in Table 5. It is noted that the comparison with the experiment confirm (i) an average relative deviation smaller than 0.4% for rotational constants and (ii) the good accuracy that rDSD is able to reach in the prediction of quadrupole coupling constants. This table also points out that the first four conformers (one of type I, one of type I’ and two of type II) are significantly more stable than the remaining ones, with the Ig${}^{-}$g conformer becoming the most stable one once moving from relative electronic energies to standard free energies. At the rDSD/B3 level (rDSD referring to the level of theory used for electronic energies, B3 to the level employed for the computation of ZPE and thermal corrections), the IIgg conformer lies 44.1 cm${}^{-1}$ above (38.7 cm${}^{-1}$ employing ChS electronic energies).

## 3. Conclusions

In this paper, we have focused on the final steps of a general strategy that aims at the accurate structural and spectroscopic characterization of flexible molecules. The main outcome of this work is that accurate geometries, spectroscopic parameters and relative energies can be obtained by the double-hybrid rev-DSD-PBEP86 functional. In particular, when combined with the jun-cc-pVTZ basis set, this DFT model is able to provide results approaching the accuracy of those issued by the well-tested ChS approaches that exploit the gold-standard CCSD(T)(-F12) method. The performance of the ChS composite schemes for geometrical parameters (and thus equilibrium rotational constants) is matched (if not improved) by rDSD when this is coupled with the linear regression approach (LRA), which does not involve any additional computational cost.

In detail, we have demonstrated that rotational and quadrupole coupling constants—parameters that strongly depend on mass distribution and intra-molecular interactions—can be obtained at the rDSD level with an accuracy which is suitable for guiding and supporting experiments in the field of rotational spectroscopy. The results obtained for glycine and serine are in very good agreement with the available spectroscopic data and pave the way toward the accurate investigation of large biomolecule building blocks.

## Figures and Tables

**Table 1:** A14 database: junChS, junChSF12 and junCCF12+CV CP-corrected interaction energies ${}^{a}$ [19]. All the values are in kJ mol^−1^.

	junChS	junChSF12	junCCF12+CV	Best ${}^{b}$
H_2_O···H_2_O	−21.10	−21.00	−21.03	−21.07
NH_3_···NH_3_	−13.30	−13.20	−12.99	−13.26
HF···HF	−19.45	−19.25	−19.25	−19.18
CH_2_O···CH_2_O	−19.23	−18.96	−18.59	−18.89
HCN···HCN	−19.88	−19.84	−19.83	−19.95
C_2_H_4_···C_2_H_4_	−4.75	−4.66	−4.36	−4.64
CH_4_···CH_4_	−2.25	−2.20	−1.99	−2.27
H_2_O···NH_3_	−27.57	−27.47	−27.34	−27.39
H_2_O···C_2_H_4_	−10.86	−10.77	−10.55	−10.82
C_2_H_4_···CH_2_O	−6.94	−6.83	−6.56	−6.84
NH_3_···C_2_H_4_	−5.89	−5.82	−5.61	−5.84
HF···CH_4_	−7.13	−7.04	−6.85	−6.95
H_2_O···CH_4_	−2.78	−2.77	−2.68	−2.85
NH_3_···CH_4_	−3.24	−3.23	−3.11	−3.26
MAX ${}^{c}$	0.34	0.11	0.30	
MUE ${}^{c}$	0.11	0.06	0.19	
RMSD ${}^{c}$	0.14	0.07	0.21	

^*a*^ Energies evaluated at the reference geometries from [81]. ^*b*^ Errors are with respect to the best values from [82]. ^*c*^ MAX, MUE and RMSD stand for maximum error, mean unsigned error, and root-mean-square deviation, respectively.

**Table 2:** Statistical analysis of the geometrical parameters, for a subset of the SE100 database, obtained at different levels of theory.

	junChS	DZCCF12	DZCCF12+CV	TZCCF12+CV	junCCF12+CV
2nd-row (17 molecules)					
MAX(r)	0.0042	0.0044	0.0027	0.0039	0.0044
MAX(θ, ϕ)	1.52	0.61	0.62	0.79	0.62
MUE(r)	0.0011	0.0022	0.0009	0.0008	0.0007
MUE(θ, ϕ)	0.19	0.19	0.16	0.16	0.15
3rd-row (7 molecules)					
MAX(r)	0.0012	0.0046	0.0040	0.0021	0.0014
MAX(θ, ϕ)	0.10	0.21	0.24	0.09	0.17
MUE(r)	0.0005	0.0018	0.0010	0.0004	0.0005
MUE(θ, ϕ)	0.05	0.10	0.09	0.02	0.06

**Table 3:** Harmonic frequencies (cm${}^{-1}$) for the molecules of the H8 set.

	DZCCF12	junCCF12	augCCF12	rDSD
MAX ${}^{a}$	13.7	12.8	18.4	18.2
MUE ${}^{a}$	4.1	4.0	4.6	5.6
RMSD ${}^{a}$	5.0	5.2	6.1	7.1

^*a*^ Statistical measures have been determined with respect to the best available values. See text for details.

**Table 4:** Equilibrium rotational constants and ^14^N-nuclear quadrupole coupling constants (MHz) of the two most stable conformers of glycine and serine. The relative energy differences (ΔE, in cm${}^{-1}$) are also reported.

	Parameter	Exp. ${}^{a}$	ChS ${}^{b}$	rDSD ${}^{b}$	rDSD-LRA ${}^{b}$	MP2/cc-pVTZ ${}^{b}$	B3LYP/SNSD ${}^{b}$
Glycine (I)	A${}_{e}$	10,418.2	10,396.6	10,334.8	10,390.3	10,328.0	10,283.1
	B${}_{e}$	3906.9	3901.1	3879.9	3897.6	3905.0	3831.1
	C${}_{e}$	2934.4	2930.4	2913.5	2927.4	2926.2	2882.9
	$\chi_{aa}$	−1.208(9)	−1.278	−1.336			
	$\chi_{bb}$	−0.343(8)	−0.464	−0.448			
	$\chi_{cc}$	1.552(10)	1.742	1.785			
Glycine (II)	A${}_{e}$	10,144.5	10,205.3	10,139.3	10,193.8	10,178.7	10,135.0
	B${}_{e}$	4094.5	4095.6	4078.7	4097.2	4104.7	4043.4
	C${}_{e}$	3024.7	3030.6	3021.3	3035.8	3041.0	2993.1
	$\chi_{aa}$	1.773(2)	1.876	1.922			
	$\chi_{bb}$	−3.194(4)	−3.286	−3.344			
	$\chi_{cc}$	1.421(4)	1.413	1.422			
	ΔE		201.5	214.8		157.2	237.8
Serine (Ig${}^{-}$g)	A${}_{e}$	4528.1	4499.4	4487.0	4510.4	4494.9	4485.2
	B${}_{e}$	1838.8	1841.3	1822.7	1831.6	1830.8	1809.1
	C${}_{e}$	1460.9	1460.0	1451.8	1459.0	1462.7	1433.3
	$\chi_{aa}$	−4.302(3)	−4.416	−4.554			
	$\chi_{bb}$	2.8236(6)	2.852	2.868			
	$\chi_{cc}$	1.479(5)	1.565	1.685			
Serine (IIgg)	A${}_{e}$	3585.9	3560.2	3559.3	3578.1	3554.8	3547.2
	B${}_{e}$	2412.7	2410.2	2393.0	2404.7	2414.0	2342.0
	C${}_{e}$	1754.4	1757.6	1739.7	1748.2	1757.9	1713.6
	$\chi_{aa}$	−3.462(2)	−3.530	−3.670			
	$\chi_{bb}$	2.0797(9)	2.149	2.134			
	$\chi_{cc}$	1.382(5)	1.381	1.536			
	ΔE		−167.2	−161.8		−185.5	−100.8

^*a*^ SE equilibrium rotational constants obtained by correcting the experimental ground-state rotational constants for computed vibrational corrections. Experimental data are from [28] for glycine and [33] for serine. Computed vibrational corrections are from [49] for glycine and [95] for serine. Experimental nuclear quadrupole coupling constants are from [28] for glycine and [33] for serine. ^*b*^ Glycine: ChS and MP2 results from [49]; rDSD and rDSD-LRA results from this work; B3LYP results from [71] and this work. Serine: ChS results from [95]; all the other data from this work.

**Table 5:** Ground-state rotational constants (A${}_{0}$, B${}_{0}$, and C${}_{0}$ in MHz) and ^14^N-nuclear quadrupole coupling constants ($\chi_{ii}$, *i* = a, b, c, in MHz) of the seven conformers of serine experimentally investigated. Computed relative standard free energies (in cm${}^{-1}$) are also reported.

	Ig${}^{-}$g	IIgg	I’gg${}^{-}$	IItg${}^{-}$	III’gg	IIg${}^{-}$t	III’tg${}^{-}$
Calc. ${}^{a}$							
A${}_{0}$	4461.34	3549.33	3505.74	3630.86	3950.32	4508.13	3464.84
B${}_{0}$	1823.01	2372.38	2305.21	2382.52	2222.91	1843.00	2304.68
C${}_{0}$	1441.95	1734.67	1803.62	1515.28	1657.03	1462.05	1604.74
$\chi_{aa}$	−4.5535	−3.6696	−0.9235	−3.8114	−0.6094	−0.3660	−1.0975
$\chi_{bb}$	2.8681	2.1341	2.5528	2.1268	−0.6702	2.0569	−0.6582
$\chi_{cc}$	1.6854	1.5355	−1.6293	1.6847	1.2796	−1.6909	1.7557
ΔG${}^{0}$	0.0	44.1	222.4	295.6	481.2	522.7	620.6
Exp. ${}^{b}$							
A${}_{0}$	4479.0320(12)	3557.20088(35)	3524.38806(41)	3638.05784(38)	3931.7548(76)	4517.473(17)	3510.4015(35)
B${}_{0}$	830.16170(25)	2380.37208(40)	2307.76826(70)	2387.89651(99)	2242.76701(70)	1846.99360(30)	2321.90829(24)
C${}_{0}$	1443.79545(28)	1740.92458(10)	1805.20788(60)	1519.18716(36)	1664.53012(57)	1463.79646(31)	1584.38608(32)
$\chi_{aa}$	−4.3023(27)	−3.4616(19)	−1.1343(35)	−3.6257(57)	−0.6733(67)	−0.6066(55)	−1.0486(55)
$\chi_{bb}$	2.82359(63)	2.07974(93)	2.5043(50)	2.06213(26)	−0.456(16)	2.0723(82)	−0.5637(53)
$\chi_{cc}$	1.4788(46)	1.3819(47)	−1.3701(50)	1.5906(50)	1.129(16)	−1.466(30)	1.612(21)

^*a*^ All the computed data are at the rDSD level (rDSD-LRA for equilibrium rotational constants) except for vibrational corrections to equilibrium rotational constants, ZPE and thermal contributions to ΔG, which are all at the B3 level. ^*b*^ Experimental data are from [33]. Standard errors are shown in parentheses in units of the last digits.

## Data Availability

Not applicable.
